# Capillary hemangioma arising from the lesser omentum in an adult: A case report

**DOI:** 10.1097/MD.0000000000018693

**Published:** 2020-01-24

**Authors:** Hideki Nagano, Takanori Goi, Seiichi Taguchi, Takayoshi Tsubaki, Toshikuni Tsuchiyama, Hidemasa Uematsu, Sakon Noriki

**Affiliations:** a Department of Surgery, Japan Community Health Care Organization Fukui Katsuyama General Hospital, Nagayama-cho; b Department of Surgery, National Hospital Organization Tsuruga Medical Center, Sakuragaoka; c First Department of Surgery, Faculty of Medicine, University of Fukui, Matsuokashimoaizuki, Eiheiji-cho, Yoshida-gun; d Department of Radiology, Japan Community Health Care Organization Fukui Katsuyama General Hospital, Nagayama-cho; e First Department of Pathology, Faculty of Medicine, University of Fukui, Matsuokashimoaizuki, Eiheiji-cho, Yoshida-gun, Japan.

**Keywords:** capillary hemangioma, computed tomography, laparoscopic surgery, lesser omentum, MIB-1 index

## Abstract

**Rationale:**

Although capillary hemangiomas, common lesions involving the proliferation of small capillary vessels and a single layer of endothelial cells, can arise in any organ, they are rarely reported in the greater or lesser omentum. Here in, we report a case of capillary hemangioma arising from the lesser omentum in an adult with interesting diagnostic imaging findings, including changes in tumor size over time on computed tomography (CT), that was resected using laparoscopic surgery. To our knowledge, this is the first English report to describe a capillary hemangioma arising from the lesser omentum.

**Patient concerns:**

A 63-year-old Japanese man received hemodialysis for chronic renal failure due to diabetic nephropathy, and a small, gradually enlarging tissue mass was found near the lesser curvature of the stomach on plain CT performed annually, without any associated complaints. Diagnostic imaging revealed an 18 × 15-mm tumor with a homogenous, highly enhanced effect in the early phase that was attenuated but prolonged in the delayed phase. Magnetic resonance imaging showed a mass with low signal intensity on T1-weighted imaging and relatively high signal intensity on T2-weighted imaging.

**Diagnosis:**

The patient was diagnosed with capillary hemangioma arising from the lesser omentum according to the pathological and immunohistological findings.

**Interventions:**

The patient underwent laparoscopy for excision of the tumor from the lesser omentum.

**Outcomes:**

At the 1 year follow-up, the patient had no recurrence of the tumor.

**Lessons:**

We describe the first case worldwide of capillary hemangioma that was a true vascular tumor arising from the lesser omentum. Although capillary hemangioma arising from the lesser omentum is extremely rare, it should be considered in the differential diagnosis of patients presenting with a highly enhanced lesser omental tumor, and laparoscopy can be safely applied for the excision of this tumor.

## 1 Introduction

Until recently, hemangiomas were thought to be due to hamartoma lesions originating from the embryonic sequestration of mesodermal tissue in any organ.^[1]^ Vascular anomalies, often named hemangiomas, include a broad spectrum of disorders, and knowledge of these disorders has increased considerably. These anomalies have been reviewed from such viewpoints as constitution, natural evolution and treatment, and attempts have been made to stratify them into lesions with a proliferative component (ie, vascular tumors) and relatively static lesions (ie, vascular malformations).^[2]^ Despite long-standing efforts to achieve a uniform classification, the nomenclature of vascular anomalies continues to be confusing.^[2]^ The key features of capillary hemangiomas remain under debate. The pathological examination of capillary hemangiomas reveals the proliferation of capillary-sized vessels and a single layer of endothelial cells. These tumors can be found in any organ, although a higher prevalence in the skin and subcutaneous tissue has been reported. They are very rare in the gastrointestinal tract^[3–5]^ and even rarer in the mesentery^[6]^ or omentum.^[7–12]^ To our knowledge, the English-language reports of hemangiomas that occurred in the greater or lesser omentum to date include only 6 cases.^[7–12]^ The 5 vascular tumors arising from the greater omentum consisted of 2 cases of capillary hemangioma, 1 hemangiopericytoma, 1 epithelioid hemangioendothelioma and 1 malignant hemangioendothelioma. The 1 case of a lesser omental tumor was a cavernous hemangioma. The 2 reported cases of capillary hemangioma were in infants.

The purpose of this article was to highlight the diagnostic imaging, including the characteristic features on computed tomography (CT), the chronological changes in tumor size, and the histopathological and immunohistochemical findings of a small capillary hemangioma arising from the lesser omentum used to determine whether it was a remnant tumor of an infantile hemangioma or a newly developed tumor. To our knowledge, this report is the first to describe a capillary hemangioma arising from the lesser omentum.

## 2 Case report

**Figure 1:**
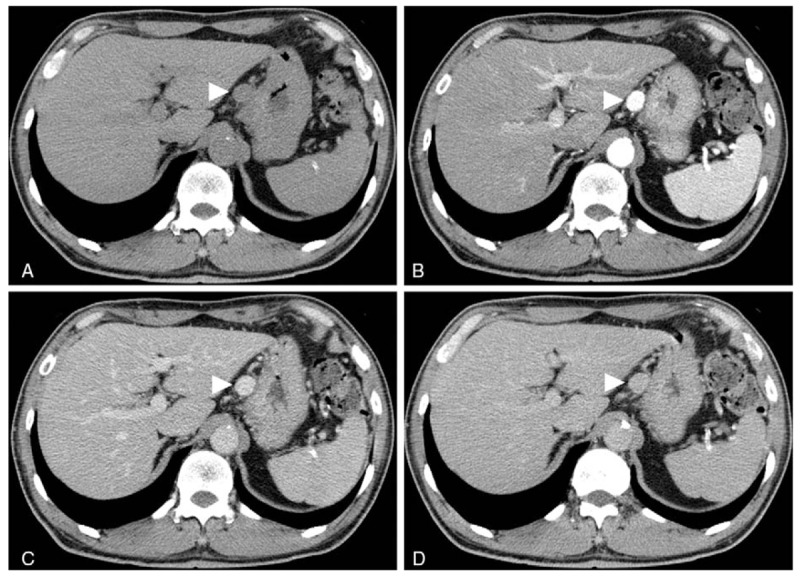
Contrast-enhanced CT revealed a round-shaped tissue mass that was 18 × 15 mm by the lesser curvature of the stomach. A: Plain CT showed a tissue mass (arrowhead) without calcification. B: The early phase of enhanced CT revealed a well-circumscribed mass (arrowhead), which showed a homogenous, highly enhanced effect without communication with the gastric wall. C: The image obtained at 120 s after the injection of contrast medium showed attenuation of the mass (arrowhead), but the contrast effect was still present. D: The image obtained at 5 min after the injection of contrast medium showed isodensity of the mass (arrowhead) compared with the liver and spleen. CT = computed tomography.

**Figure 2:**
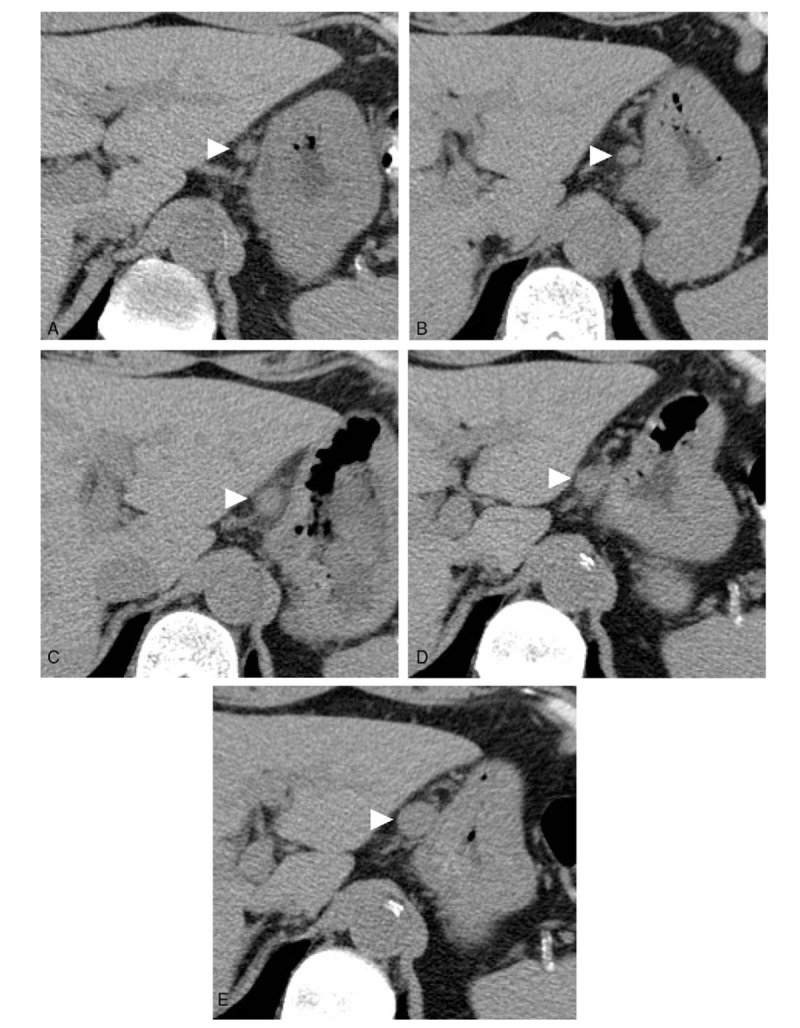
Gradual enlargement of the mass as shown by annual plain CT. The mass of interest (arrowhead) measured 10 × 8 mm in diameter in June 2013 (A), 10 × 9 mm in July 2014 (B), 16 × 10 mm in July 2015 (C), 16 × 11 mm in June 2016 (D), and 18 × 14 mm in June 2017 (E). These images show a gradual and clear increase in the tumor size. CT = computed tomography.

**Figure 3:**
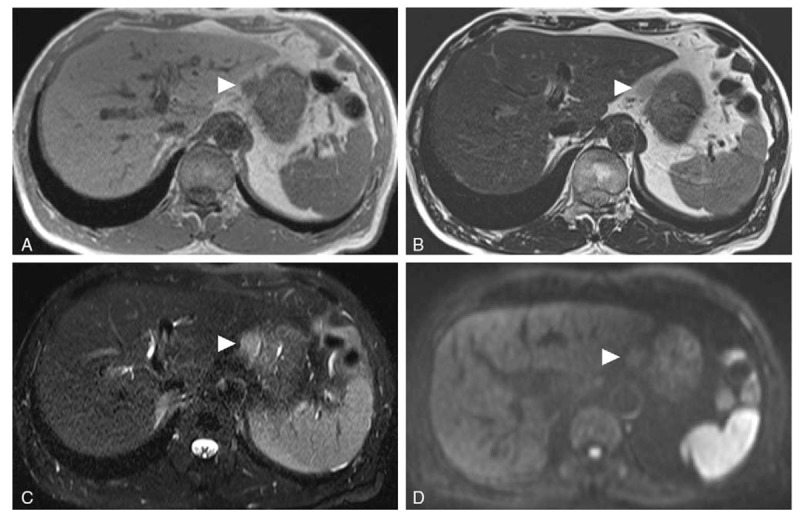
Representative MRI images of a capillary hemangioma located in the lesser omentum. A: T1WI in the axial plane showing a mass (arrowhead) by the lesser curvature with low SI equivalent to that in the muscles or spleen. B: T2WI showing relatively high SI of the mass (arrowhead). C: Fat-suppressed T2WI showing no decrease in the SI of the mass (arrowhead). D: Diffusion-weighted MRI showing isointensity of the mass (arrowhead) compared with the liver. MRI = magnetic resonance imaging, SI = signal intensity, T2WI = T2-weighted imaging.

**Figure 4:**
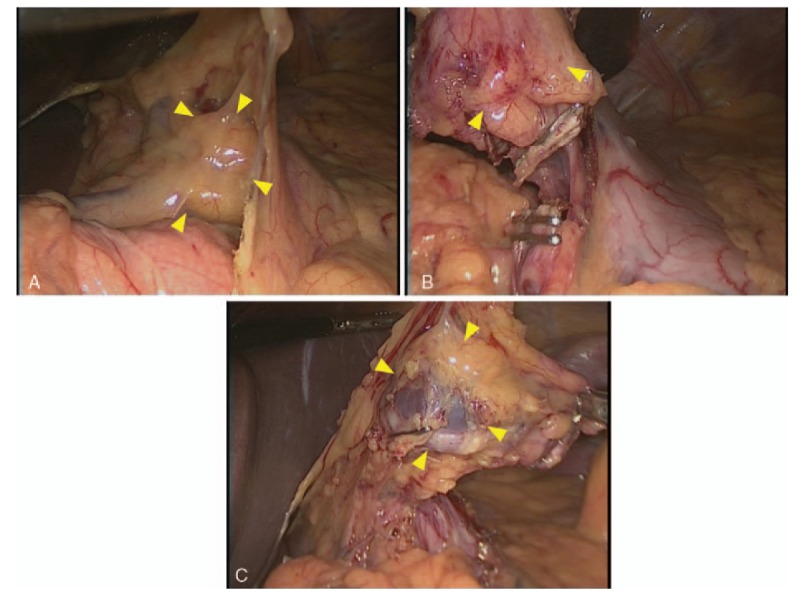
Surgical findings. A dark red, elastic, soft tumor was located in the lesser omentum without contact with the stomach wall. The tumor was surrounded by the peritoneum of the lesser omentum and was fed from some branches of the left gastric artery (A). The tumor was removed easily by laparoscopic surgery (B, C).

A 63-year-old Japanese man with chronic renal failure due to diabetic nephropathy was treated with hemodialysis in the Department of Internal Medicine at the Japan Community Health Care Organization Fukui Katsuyama General Hospital. A gradually enlarging tissue mass was identified as a lymph node by the lesser curvature of the stomach on plain CT in June 2017. The patient had already undergone an endoscopic examination of the upper digestive tract in February of that year and was diagnosed with chronic atrophic gastritis, but no neoplastic lesions had been identified. The patient, who had no complaints, was referred to a gastroenterologist in July 2017. He underwent an enhanced CT study of the abdomen with a venous bolus injection of contrast medium. A tissue mass that measured 18 × 15 mm was observed adjacent to the lesser curvature of the stomach, and there was no communication with the gastric wall. Enhanced CT showed homogeneous, high enhancement of the tissue mass in the early phase that was attenuated but prolonged after 120 seconds (Fig. 1). The findings of predominant enhancement during the arterial phase suggested the possibility of a paraganglioma, a solitary aneurysm, an extragastrointestinal stromal tumor (EGIST), Castleman disease, a solitary fibrous tumor, or a splenotic nodule. Based on the high and homogenous enhancement pattern, the location of the tissue mass, and the lack of a past history of trauma, we suspected a paraganglioma, an aneurysm, or an EGIST. Annual plain abdominal CT had been performed since 2013, and we reviewed all of these previous scans. The soft tissue nodule of interest measured 10 × 8 mm in diameter in June 2013, 10 × 9 mm in July 2014, 16 × 10 mm in July 2015, 16 × 11 mm in June 2016, 18 × 14 mm in June 2017, and 18 × 15 mm in February 2018 (Fig. 2). These images revealed a gradual and obvious increase in the tumor size. Magnetic resonance imaging (MRI) of the abdomen showed a well-circumscribed mass by the lesser curvature of the stomach with low signal intensity (SI) equivalent to that in the muscles or spleen on T1-weighted imaging (T1WI), relatively high SI on T2-weighted imaging (T2WI) and no drop in SI on out-of-phase T2WI (Fig. 3). These findings exclude the possibility of an aneurysm because of the absence of a flow void; rather, the anomaly was considered a tumor without a fat component. The patient underwent ^123^I metaiodobenzylguanidine (^123^I-MIBG ) scintigraphy at the University of Fukui Hospital, but abnormal accumulation of the isotope was not detected. He was referred to our department for surgical resection of the enlarging tumor and a definitive diagnosis. The patient had a medical history of diabetic nephropathy and chronic renal failure. On physical examination, the patient had a height of 170 cm, a body weight of 61.5 kg, a pulse of 65 beats/min, a blood pressure of 117/77 mm Hg, and a body temperature of 36.8°C. Anemia, jaundice, edema, and malnutrition were not found. No abnormalities were detected in the skin or subcutaneous tissue. Superficial lymph nodes were not detected on palpation. His abdomen was flat and soft, and no mass was detected on palpation. Routine laboratory tests on the day after dialysis showed leukopenia (white blood cell count, 2,900/μL), anemia (red blood cell count, 3.85 × 10^6^/mL; hemoglobin, 12.6 g/dL; hematocrit, 39.5%) and renal dysfunction (blood urea nitrogen, 33.2 mg/dL; creatinine, 8.65 mg/dL). The plasma levels of vanillylmandelic acid (VMA; 48.2 ng/mL, normal range, 3.3–8.6 ng/mL) and homovanillic acid (HVA; 22.2 ng/ml, normal range, 4.4–15.1 ng/mL) were elevated. Considering the imaging results and the chronic enlargement of the tissue mass, we made a preoperative diagnosis of paraganglioma in the lesser omentum as the most likely diagnosis even though we were not able to reach a definitive diagnosis. The patient underwent laparoscopic surgery in February 2018 (Fig. 4). A dark red, elastic, soft tumor was located in the lesser omentum without extension to the stomach wall. The tumor was surrounded by the peritoneum of the lesser omentum and was fed by some branches of the left gastric artery. After placing clips on the feeding vessels, the tumor was easily resected. During the operation, no remarkable changes in blood pressure or heart rate were observed.

### 2.1 Pathological findings

**Figure 5:**
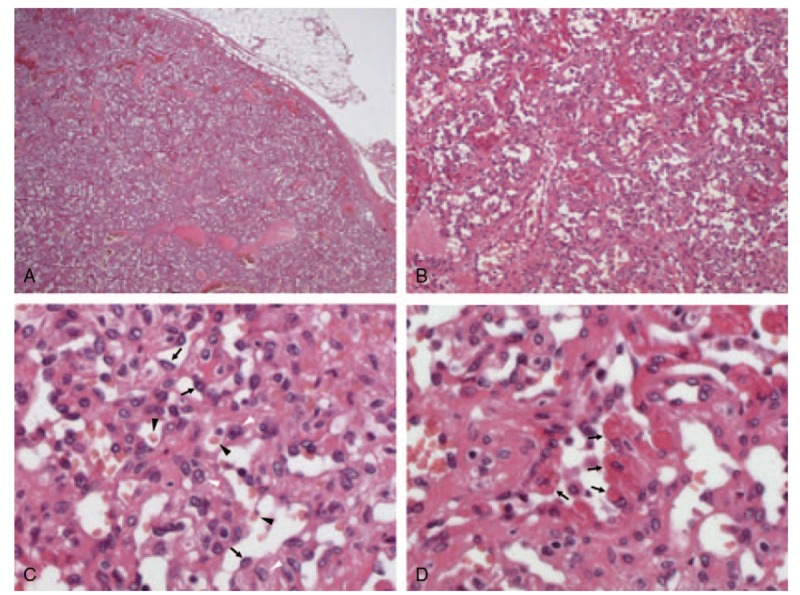
Histopathological findings. The tumor was well demarcated (A: × 20) and composed of well-differentiated blood vessels containing both endothelial cells (arrow) and pericytic elements (white arrowhead) (B: × 100. C: × 400). The capillary lumens had erythrocytes (black arrowhead) (C). Some endothelial cells showed hemophagocytosis (arrow) (D: × 400). The tissues were subjected to hematoxylin-eosin staining.

**Figure 6:**
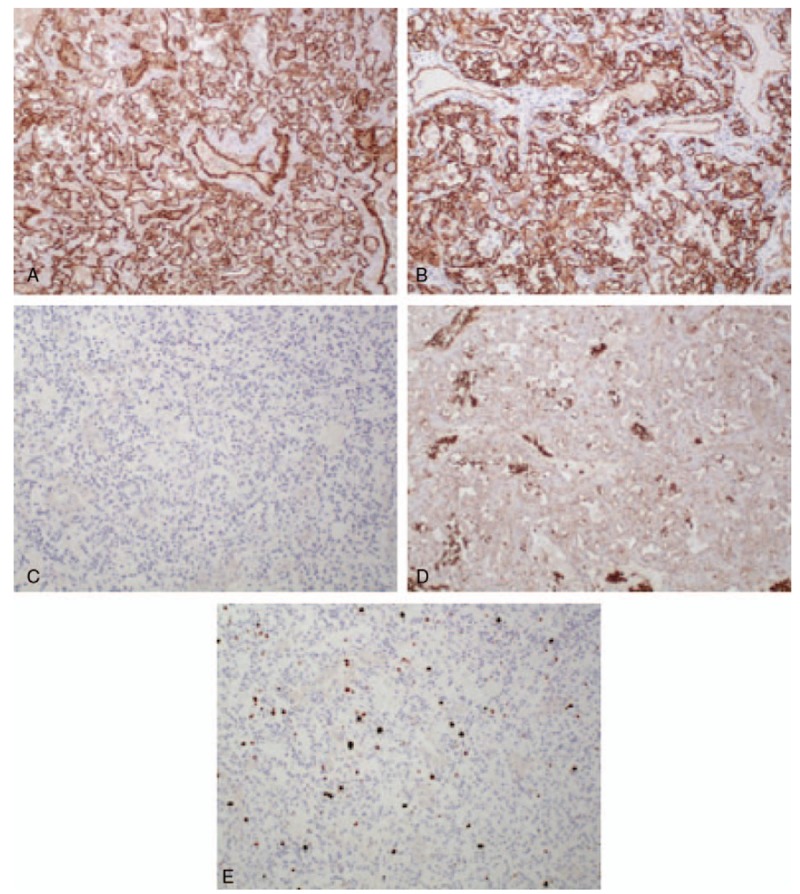
Immunohistological findings. Tumor cells were positive for the vascular markers CD31 (A: × 100) and CD34 (B: × 100) but negative for the lymphatic marker D2-40 (C: × 100). GLUT-1, which is a positive marker for infantile hemangioma and perineural cells, was negative in tumor cells but positive in erythrocytes (D: × 100). The MIB-1 index, which indicates the proliferative capacity of the tumor, was 8.5% (E: × 100). The tumor was diagnosed as a capillary hemangioma. CD = cluster of differentiation, GLUT-1 = glucose transporter 1.

Macroscopically, the resected specimen showed a dark red, smooth surface with a thin capsule. The tumor was soft and elastic, measuring 15 × 13 mm, and it was well demarcated. Histological examination revealed that the tumor had well-differentiated blood vessels containing endothelial cells (Fig. 5A-C), which were positive for cluster of differentiation (CD) 31 and CD 34 (Fig. 6A and B) but negative for D2–40, glucose transporter 1 (GLUT-1) (Fig. 6C and D), vascular endothelial growth factor A, insulin-like growth factor 2 (IGF-2) and pericytic elements. The endothelial cells showed hemophagocytosis (Fig. 5D). The tumor cells had no nuclear atypia, and there were no malignant findings. Regarding the proliferative capacity of the tumor, the molecular immunology Borstel 1 (MIB-1) index was 8.5% (Fig. 6E). The tumor was diagnosed as a capillary hemangioma based on these findings.

The patient showed an uneventful recovery, and he was discharged from the hospital 4 days after the operation. At the 1-year follow-up, the patient had no recurrence of the tumor.

## 3 Discussion

Vascular anomalies of the gastrointestinal tract are rare and account for < 2% of all benign gastric neoplasms^[13]^ and 2.8% of all intestinal neoplasms.^[14]^ Our review of the English literature showed reports of these anomalies occurring in the greater omentum in only 5 previous cases,^[7–11]^ and there has been only one report of a hemangioma arising from the lesser omentum.^[12]^ The 5 vascular tumors arising from the greater omentum consisted of 2 cases of capillary hemangioma, one hemangiopericytoma, 1 epithelioid hemangioendothelioma and 1 malignant hemangioendothelioma. The 1 case of a lesser omental tumor was a cavernous hemangioma. The 2 reported cases of capillary hemangioma were in infants. Our case, to our knowledge, is the first report of a capillary hemangioma arising from the lesser omentum. After the patient was referred for surgery, we reviewed the annual abdominal plain CT scans from June 2013 to June 2017. Because the plain CT scans initially focused on renal function and morphological changes, CT with contrast was not performed. The small nodule seen on plain CT was initially diagnosed as nonspecific lymphadenopathy. Since an increase in the size of the tumor was observed slowly over time, we performed CT with contrast. Contrary to our previous expectation of a diagnosis of lymphadenopathy, the tumor showed a strong homogenous contrast effect in the early phase of enhanced CT. The contrast effect gradually decreased but was prolonged compared with that in the aorta in the same phase. Based on these findings, we considered the possibility of a paraganglioma, a solitary aneurysm, or an EGIST^[15]^; however, we could not establish a definitive preoperative diagnosis.

The reported characteristic findings of a hemangioma on dynamic enhanced CT include peripheral enhancement during the dynamic bolus phase and complete isoattenuated fill-in during the delayed phase for up to 60 minutes after the administration of contrast medium.^[16]^ However, in another report, a small subset of hemangiomas (3.8% or 12.3%, based on 2 different readers) showed a contrast density that was similar to that in the aorta during the arterial phase, as observed in the present case.^[17]^ Kim et al^[18]^ found that small hemangiomas (<2 cm) had marked enhancement in the early phase on enhanced CT, but this effect dissipated in the delayed phase. Findings similar to those described by Kim et al^[18]^ were observed in our case. In contrast to tumors in the lesser omentum, a paraganglioma shows a well-demarcated, solid, cystic, heterogeneously enhanced mass on enhanced CT.^[19,20]^ However, no CT features unique to paraganglioma have been found, despite the tendency for smaller tumors to be more homogenous and have more well-defined margins than larger tumors.^[20]^ The laboratory tests showed elevated plasma levels of VMA and HVA despite the final diagnosis of capillary hemangioma. Iwamoto et al^[21]^ showed that the plasma VMA level was elevated in patients on hemodialysis both with and without pheochromocytoma. In patients on hemodialysis, it seems that the plasma level of catecholamine metabolites is not appropriate as an index for the diagnosis of paraganglioma. Although ^123^I-MIBG scintigraphy in our case did not show isotope accumulation, close attention should be paid to ^123^I-MIBG-negative cases, as this result is found rather frequently in cases of metastatic pheochromocytoma.^[22]^

Although the MRI findings in our case, such as low SI on T1WI and relatively high SI on T2WI, did not allow a specific diagnosis to be made, it has been reported that venous malformations typically show uniformly high SI on T2WI, whereas capillary hemangiomas tend to be less intense because of shrinkage due to different degrees of fibrosis and hemosiderin deposition.^[10,12]^ Characteristic T2WI findings would be useful for discriminating between capillary hemangiomas and other venous anomalies, and the findings in our case are consistent with a capillary hemangioma.^[23]^ Pathologically, hemangiomas have previously been classified according to channel size into 3 types, namely, cavernous, capillary, or mixed, and the cavernous type is the most common.^[24]^ Hemangiomas can be classified by different parameters:

(1) Vessel type (eg, capillary, cavernous, venous);
(2) Location (eg, cutaneous, intramuscular, synovial);
(3) Characteristic cell type (eg, epithelioid, spindle cell, hobnail);
(4) Neoplastic status (eg, true neoplasia, vascular malformation, telangiectasia, reactive hyperplasia); and
(5) Patient age (eg, juvenile, senile).

Capillary hemangiomas are commonly considered congenital, true vascular tumors that usually tend to involute during childhood, and only a minority persist throughout adulthood.^[25]^ Unlike the 2 previously reported cases of capillary hemangioma in the omentum, our case occurred in adulthood. Furthermore, a gradual increase in size occurred over 4 years, and there was negative immunostaining for GLUT-1, which is a downstream target of hypoxia-inducible factor 1 alpha.^[26]^ There was also negative immunohistochemical reactivity for VEGF-A and IGF-2, which are sensitive markers for the diagnosis of infantile hemangioma.^[27]^ These findings suggest that the tumor was not a residual anomaly of a congenital hemangioma but rather that it developed in adulthood. The MIB-1 index of the tumor suggests that it was a neoplastic lesion.

Surgical excision should be considered for the treatment of capillary hemangiomas in the lesser omentum, and pathological examination of the resected tissue can be used to obtain a definitive diagnosis. Furthermore, laparoscopic surgery seems to be effective for the excision of hemangiomas in the lesser omentum following thorough preoperative evaluations of the diagnostic images to assess the tumor size, anatomical location, extent of invasion into the gastric wall and laparoscopic surgical field, as this technique has the advantages of minimal surgical invasiveness, reduced wound healing time, reduced postoperative pain, reduced blood loss, reduced wound-related complications and improved cosmetic outcomes.^[28]^

## 4 Conclusion

We report an extremely rare case of capillary hemangioma arising from the lesser omentum in adulthood. Although capillary hemangioma of the lesser omentum is an extremely rare disease, it should be considered in the differential diagnosis of patients presenting with a highly enhanced lesser omental tumor, and laparoscopic surgery is a good treatment option for excision of this tumor.

## Author contributions

**Conceptualization**: Hideki Nagano

**Data curation:** Hideki Nagano, Seiichi Taguchi, Takayoshi Tsubaki

**Investigation:** Seiichi Taguchi, Takayoshi Tsubaki, Toshikuni Tsuchiyama, Hidemasa Uematsu, Sakon Noriki

**Methodology:** Hideki Nagano

**Project administration:** Hideki Nagano, Seiichi Taguchi

**Supervision:** Takanori Goi

**Data curation:** Hidemasa Uematsu, Sakon Noriki.

**Supervision:** Takanori Goi, Seiichi Taguchi, Takayoshi Tsubaki, Toshikuni Tsuchiyama, Hidemasa Uematsu, Sakon Noriki.

**Validation:** Takanori Goi, Sakon Noriki.

**Writing – original draft:** Hideki Nagano.

Hideki Nagano orcid: 0000-0003-0524-2247.
